# miR-134-5p inhibits osteoclastogenesis through a novel miR-134-5p/Itgb1/MAPK pathway

**DOI:** 10.1016/j.jbc.2022.102116

**Published:** 2022-06-09

**Authors:** Meng Huang, Yan Wang, Zhenning Wang, Qiaozhen Qin, Heyang Zhang, Shuirong Liu, Jiantong Cui, Yu Zhang, Xiaoxia Jiang, Lulu Xu

**Affiliations:** 1 Medical School of Chinese PLA, Beijing, China; 2 Department of Orthodontics, The First Medical Center, Chinese PLA General Hospital, Beijing, China; 3 Beijing Institute of Basic Medical Sciences, Beijing, China

**Keywords:** osteoclastogenesis, miR-134-5p, Itgb1, MAPK, α-MEM, minimum essential medium eagle alpha modifications, BMM, bone marrow macrophage, CCK-8, cell counting kit-8, cDNA, complementary DNA, DAPI, 4′,6-diamidino-2-phenylindole, KEGG, Kyoto Encyclopedia of Genes and Genomes, micro-CT, microcomputed tomography, NC, negative control, OVX, ovariectomized, qRT-PCR, quantitative RT-PCR

## Abstract

Osteoporosis affects approximately 200 million people and severely affects quality of life, but the exact pathological mechanisms behind this disease remain unclear. Various miRNAs have been shown to play a predominant role in the regulation of osteoclast formation. In this study, we explored the role of miR-134-5p in osteoclastogenesis both *in vivo* and *in vitro*. We constructed an ovariectomized (OVX) mouse model and performed microarray analysis using bone tissue from OVX mice and their control counterparts. Quantitative RT-PCR data from bone tissue and bone marrow macrophages (BMMs) confirmed the decreased expression of miR-134-5p in OVX mice observed in microarray analysis. In addition, a decrease in miR-134-5p was also observed during induced osteoclastogenesis of BMMs collected from C57BL/6N mice. Through transfection with miR-134-5p agomirs and antagomirs, we found that miR-134-5p knockdown significantly accelerated osteoclast formation and cell proliferation and inhibited apoptosis. Furthermore, a luciferase reporter assay showed that miR-134-5p directly targets the integrin surface receptor gene *Itgb1*. Cotransfection with Itgb1 siRNA reversed the effect of the miR-134-5p antagomir in promoting osteoclastogenesis. Moreover, the abundance levels of MAPK pathway proteins phosphorylated-p38 (p-p38) and phosphorylated-ERK (p-ERK) were significantly increased after transfection with the miR-134-5p antagomir but decreased after transfection with the miR-134-5p agomir or Itgb1 siRNA, which indicated a potential relationship between the miR-134-5p/Itgb1 axis and the MAPK pathway. Collectively, these results revealed that miR-134-5p inhibits osteoclast differentiation of BMMs both *in vivo* and *in vitro* and that the miR-134-5p/Itgb1/MAPK pathway might be a potential target for osteoporosis therapy.

Osteoporosis is a widely concerning disease affecting a population of approximately 200 million that interferes with the patients’ quality of life and subsequently imposes burdens on their families and society (1). In pathological settings, patients with osteoporosis display an uncontrolled erosion of bone mass, a deteriorated microarchitecture, and bone fracture with an unbalanced microenvironment (2). The homeostasis of a healthy bone microenvironment is mainly maintained by osteoclastic bone resorption and osteoblastic bone formation (3, 4, 5, 6). However, the pathogenetic process of osteoporosis may be accompanied by excessive osteoclast activation and osteoblast reduction, which attenuates both bone mass and the normal structure of trabecular and cortical bone (7, 8, 9, 10). Therefore, it is vital to determine the underlying mechanism of abnormally activated bone resorption and identify effective methods to cure aggravating osteoporotic disease.

Osteoclasts are large multinucleated cells originating from monocyte/macrophage precursor cells. Their differentiation requires various factors, among which macrophage colony-stimulating factor (M-CSF) and receptor activator of nuclear factor kappa B ligand (RANKL) are prominent (11). After their recruitment from the bone marrow, macrophages reside on the bone surface and differentiate into multinucleated osteoclasts with the cooperation of M-CSF and RANKL. These cells can degrade the bone structure by secreting acids and proteinase to dissolve organic matrix following the formation of bone resorption lacunae (12). During this demineralization process, various cytokines and key molecules are activated in a pathological condition that manipulates bone metabolism, which may explain an effective treatment strategy for osteoporosis.

miRNAs are conserved, small noncoding RNAs with a length of approximately 22 to 24 nt that act as negative regulators of downstream gene expression (13). An extensive body of evidence shows that miRNAs participate in various biological activities, including osteoclastogenesis (14). miRNAs can diminish the bone mass by targeting some key molecules that play a critical role in osteoclast formation. Recent studies have shown that miR-506-3p inhibits osteoclasts by targeting the NFATc1 transcription factor and degrading RANKL-induced osteoclastogenesis (15). Guo *et al.* (16) demonstrated that miR-125a suppresses osteoclast differentiation through the stimulation of M-CSF and RANKL. However, miR-214 aggravates bone resorption by targeting TNFR-associated factor (TRAF)3 to activate the NF-κB pathway (17). These results indicate that miRNAs might be crucial regulatory factors of osteoclastogenesis. miR-134-5p reportedly plays a pivotal role in the repression of tumor invasion and participates in depression and Alzheimer’s disease. Moreover, a recently published study noted the auxoaction of miR-134-5p in inducing calcium deposition in vascular smooth muscle cells in rats (18, 19, 20), which has been related to matrix deposition in bone formation. However, a well-defined role for miR-134-5p in bone resorption is not completely clear.

In the present study, to better understand the effects of miR-134-5p on bone resorption, we utilized bone marrow macrophages (BMMs) of mice for the detection of osteoclast formation. We found that miR-134-5p knockdown promoted osteoclast differentiation both *in vivo* and *in vitro*. Additionally, bioinformatic websites and protocols demonstrated that miR-134-5p regulated osteoclast differentiation by directly targeting integrin β1 (Itgb1)-mediated mitogen-activated protein kinase (MAPK) pathway activation. Our study revealed that the miR-134-5p/Itgb1 axis contributes to osteoclastogenesis and bone metabolism and might thus provide clear evidence for the mechanism of bone remodeling.

## Results

### Construction of an ovariectomized mouse model

**Figure 1:**
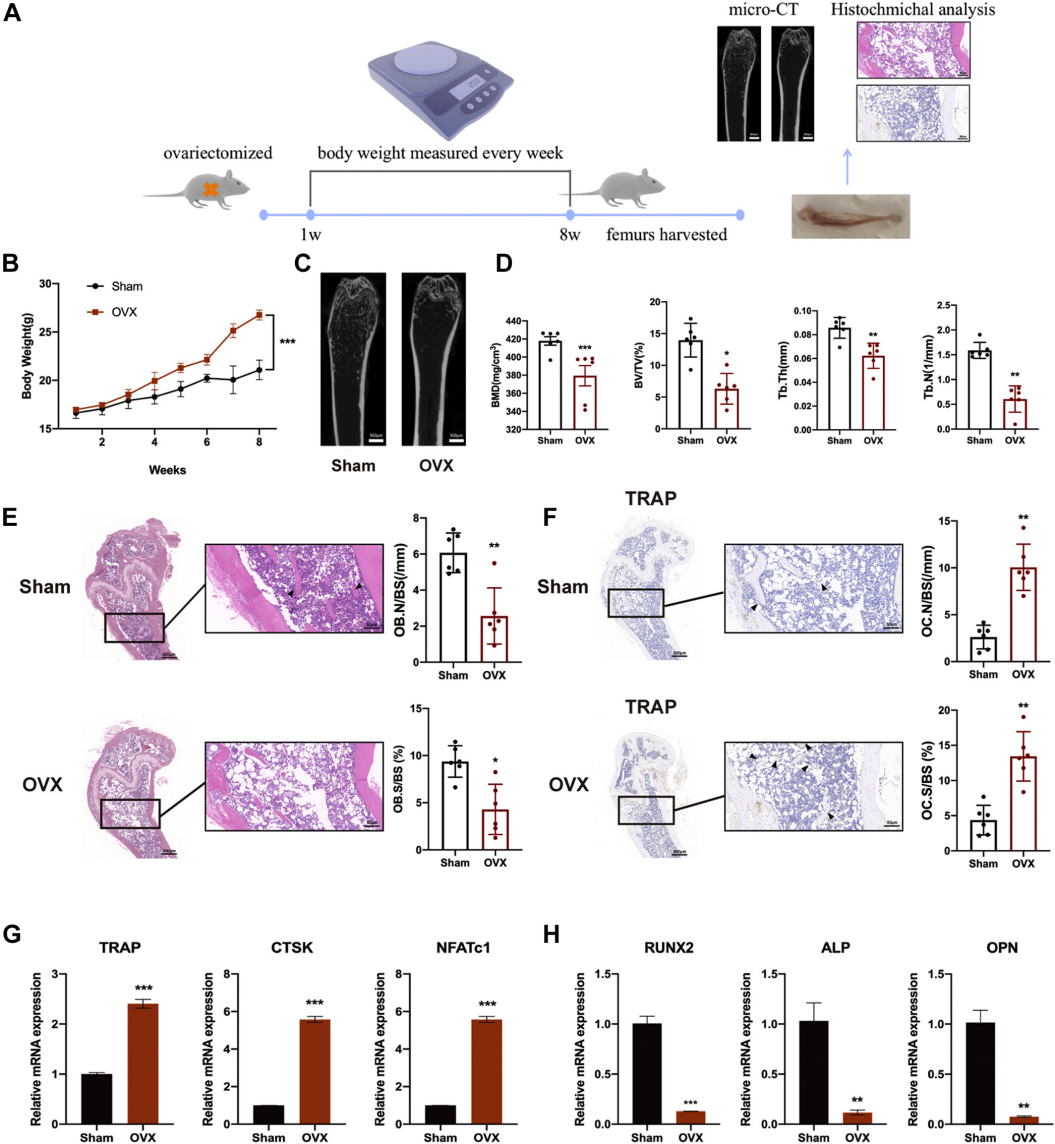
**Construction of an ovariectomized (OVX) mouse model.***A*, schematic representation of the construction of an OVX mouse model for further investigation (n = 6). The experiments performed on the construction of OVX mouse model were included and some of the images such as *C*, *E*, and *F* were shown in the schematic representation. *B*, body weights of the sham and OVX groups at 2, 4, 6, and 8 weeks. *C*, the right femurs of all the mice were collected and subjected to micro-CT scanning and images were captured. The scale bars represent 500 μm. *D*, the bone mineral density (BMD), bone volume to tissue volume (BV/TV, %), trabecular thickness (Tb. Th, mm), and trabecular number (Tb. N, 1/mm) of each sample were measured. *E* and *F*, histological analysis with H&E and immunohistochemical staining with TRAP were conducted using thigh bone tissue slices of sham and OVX mice. OB.N/BS(/mm), OB.S/BS (%), OC.N/BS(/mm), and OC.S/BS (%) were measured with ImageJ software. The scale bars for main panels represent 200 μm. The scale bars for the zoom magnification panels represent 50 μm. *G* and *H*, the expression levels of the osteoclast-related genes TRAP, CTSK, and NFATc1 and, the osteoblast-related genes RUNX2, ALP, and OPN, in bone tissues from each group were measured by qRT-PCR. β-Actin was used as an internal reference gene. All the data are expressed as the means ± SDs. ∗*p* < 0.05, ∗∗*p* < 0.01, ∗∗∗*p* < 0.001. micro-CT, microcomputed tomography; qRT-PCR; quantitative RT-PCR.

To explore the osteoclast formation process *in vivo*, we first constructed a mouse model of ovariectomy. The body weight of ovariectomized (OVX) mice was significantly higher than that of sham mice, illustrating that an increase in adipose tissue accounted for osteoporosis development (Fig. 1*B*). Images of microcomputed tomography (micro-CT) revealed significant bone loss in OVX mice, as was particularly demonstrated by a reduced trabecular amount, whereas more trabecular bone was observed in sham mice (Fig. 1*C*). Furthermore, an evaluation of cancellous bone metabolism of femurs of each group, including the bone mineral density, bone volume to tissue volume (%), trabecular thickness (millimeter), trabecular number (1/mm) was shown in Figure 1*D*. All parameters revealed that OVX mice exhibited a significant increase in bone mass loss compared with those in the sham group. A histological analysis with H&E and immunohistochemical staining with tartrate-resistant acid phosphatase (TRAP), a critical marker of osteoclast formation, were performed. As shown by H&E staining, OVX mice exhibited fewer trabecule and a higher increasing number of lipid droplets than sham mice (Fig. 1*E*). The OVX mice also exhibited a lower osteoblast number or osteoblast surface, which indicated deterioration of the underlying osteogenesis process. Additionally, the number of osteoclasts and the osteoclast surface around the trabecular bone microarchitecture were increased in OVX mice, as depicted by immunohistochemical staining (Fig. 1*F*). In addition, the mRNA expression levels of the osteoclast-related genes TRAP, CTSK, and NFATc1 in bone tissue were determined because these factors play a major role in osteoclast development. The levels of TRAP, CTSK, and NFATc1 were upregulated in OVX mice (Fig. 1*G*). In contrast, the expression of osteoblast-related genes, including RUNX2, ALP, and OCN, was downregulated, indicating the inhibition of osteogenesis (Fig. 1*H*).

### miR-134-5p was decreased in the OVX mouse model

**Figure 2:**
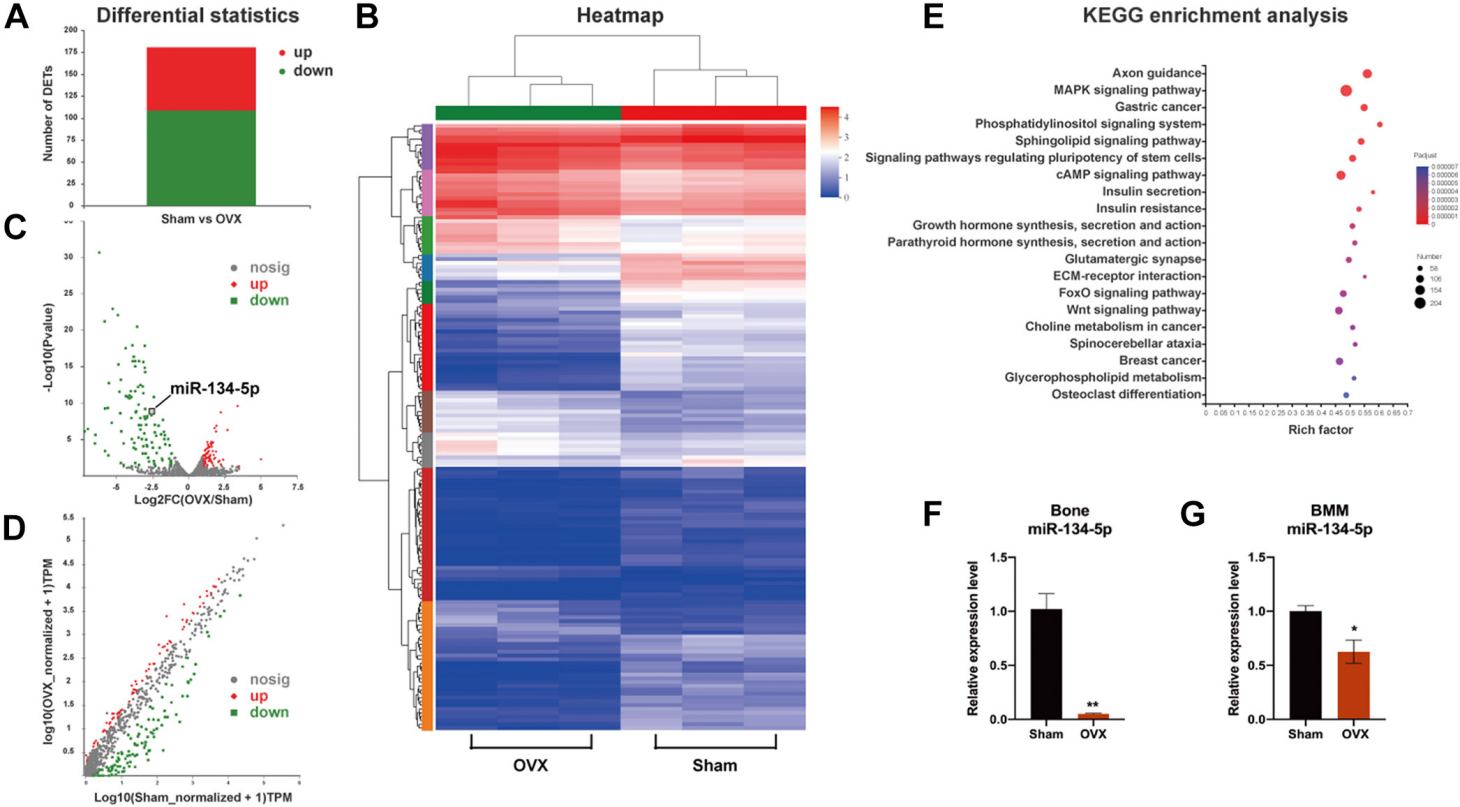
**miR-134-5p expression was decreased in an ovariectomized (OVX) mouse model.***A*, microarray profiling analysis was performed, and 181 significantly differentiated miRNAs were discovered in the samples (|log2FC| ≥ 1 and *p* value < 0.05). *B*, heatmap displaying the differentially expressed miRNAs in the samples of sham and OVX mice. *C*, volcano plot diagram showing the significantly upregulated or downregulated miRNAs. *D*, scatter plot diagram revealing the correlation among the significantly differentially expressed miRNAs. *E*, a Kyoto Encyclopedia of Genes and Genomes (KEGG) enrichment analysis was performed to determine the top related pathways involving these differentially expressed miRNAs. *F* and *G*, the expression level of miR-134-5p in bone tissue and in BMMs of the sham and OVX groups was determined. U6 was used as an internal reference gene. All the data are expressed as the means ± SDs. ∗*p* < 0.05, ∗∗*p* < 0.01. BMMs, bone marrow macrophages.

Because miRNAs play an integral role in human osteoporosis (21), we detected the expression profile of miRNAs in sham and OVX mice. Total RNA including miRNAs was extracted and subjected to microarray analysis. The microarray analysis revealed a total of 1186 miRNAs, and 181 of these miRNAs were differentially expressed (|log2FC| ≥ 1 and *p* value < 0.05) (Fig. 2*A*). As shown in the heatmap, ovariectomy upregulated and downregulated the expression of 72 and 109 miRNAs, respectively, in mice (Fig. 2*B*). Using *p* values and expression fold values, we created volcano plots to visualize the miRNAs showing differential expression between sham and OVX mice. We constructed volcano plots with *p* values and fold change values (log2FC) to indicate the relationship between fold change in expression and statistical significance. In the diagram, the *red* plots reveal significantly upregulated miRNAs, the *green* plots indicate downregulated miRNAs, and the *gray* plots indicate miRNAs that are not significantly expressed (Fig. 2*C*). We also used scatter plots to interpret the differences in miRNAs between two comparative samples. The values of the *x*-axis and *y*-axis represented in the scatter plots indicate the normalized fold change values of miRNAs from sham and OVX mice. The *red* spots at the top or the *green* spots at the bottom in the scatter plots represent upregulated or downregulated miRNAs with a fold change >1, respectively (Fig. 2*D*). A Kyoto Encyclopedia of Genes and Genomes (KEGG) enrichment analysis revealed that the differentially expressed miRNAs were enriched in the regulation of osteoclast differentiation (Fig. 2*E*). Bone tissue or BMMs from sham and OVX mice was collected, and decreased expression of miR-134-5p was detected in OVX mice by quantitative RT-PCR (qRT-PCR), confirming the results for miR-134-5p obtained in the microarray analysis (Fig. 2, *F* and *G*).

### miR-134-5p was decreased during osteoclastogenesis *in vitro*

**Figure 3:**
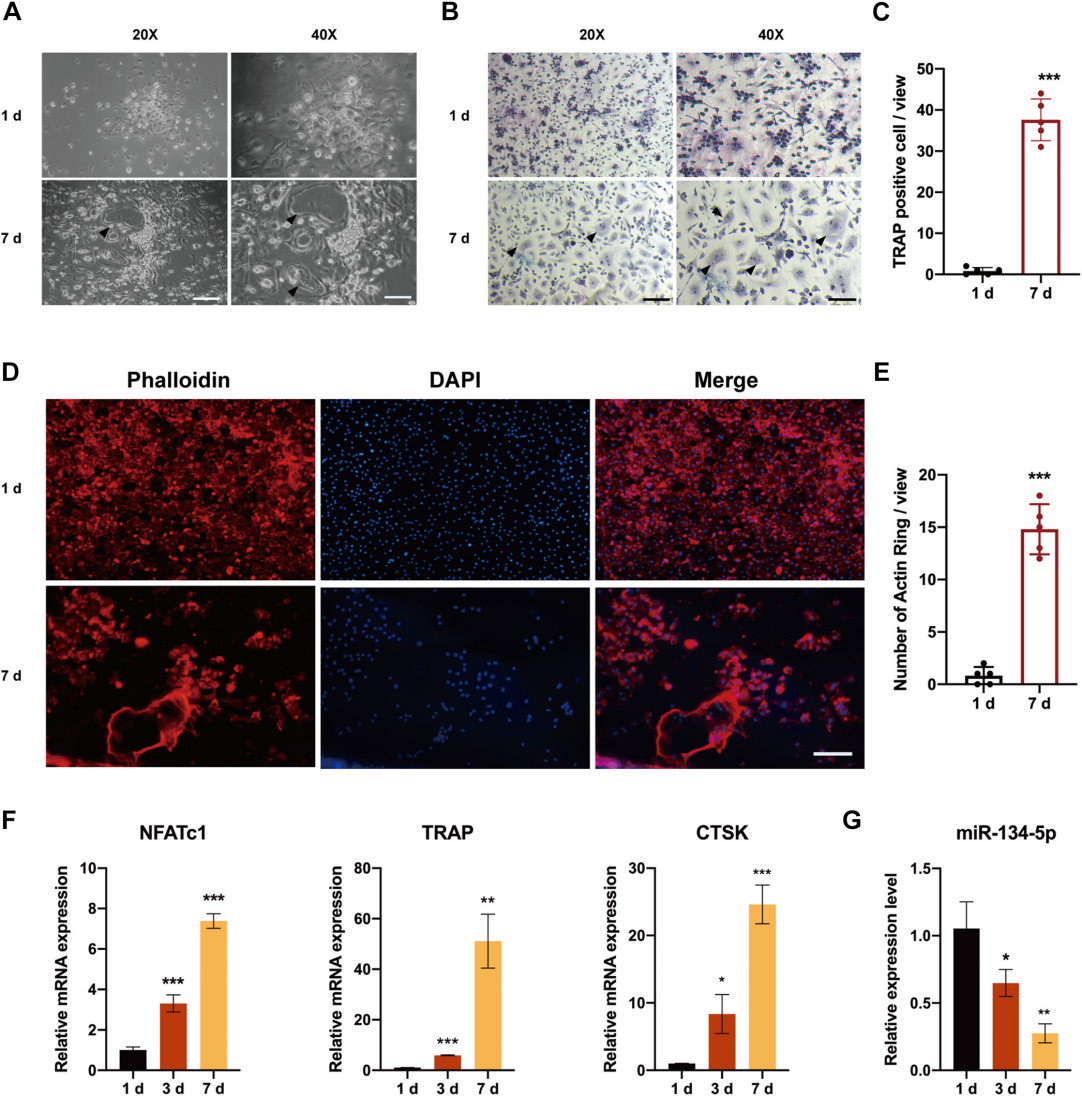
**miR-134-5p expression is decreased during osteoclast differentiation.***A*, BMMs were cultured in the presence of 30 ng/ml M-CSF and 50 mg/ml RANKL for 7 days and were identified under a light microscope. Scale bars: 20×: 100 μm; 40×: 50 μm. *B* and *C*, TRAP staining was performed to analyze osteoclast activity. The number of TRAP+ multinuclear osteoclasts was counted. The scale bars represent 20×: 100 μm; 40×: 50 μm. *D* and *E*, BMMs were induced to differentiate into osteoclasts and stained for actin ring formation using phalloidin. The number of F-actin rings per field was counted. The scale bars represent 100 μm. *F*, the mRNA expression levels of TRAP, CTSK, and NFATc1 were analyzed by quantitative RT-PCR at 1, 3, and 7 days. β-Actin was used as an internal reference gene. *G*, the expression level of miR-134-5p was analyzed by quantitative RT-PCR after induction for 1, 3, and 7 days. U6 was used as an internal reference gene. All the data were expressed as the means ± SDs. ∗*p* < 0.05, ∗∗*p* < 0.01, ∗∗∗*p* < 0.001. BMMs, bone marrow macrophages.

For the *in vitro* study, we examined the exact impact that miR-134-5p exerted on BMMs in response to M-CSF and RANKL. Initially, we induced BMMs to differentiate into mature osteoclasts by incubation with 30 ng/ml M-CSF and 50 ng/ml RANKL for 7 days. The mature multinucleated cells were then identified under a light microscope (Fig. 3*A*). Following TRAP staining, a method for measuring osteoclast-related enzyme activities, these TRAP-positive cells were identified as multinucleated osteoclasts. TRAP-positive osteoclasts were observed after induction for 7 days, whereas in the group induced for 1 day, almost no osteoclasts were observed (Fig. 3, *B* and *C*). Actin ring formation of osteoclasts was observed, and the number was counted by staining with rhodamine-phalloidin (*red*) and 4′,6-diamidino-2-phenylindole (DAPI) (*blue*). After 7 days of stimulation, the number of actin rings that had formed, as indicated by phalloidin, was increased (Fig. 3, *D* and *E*). For further confirmation, qRT-PCR analysis was performed to reveal the mRNA expression levels of TRAP, CTSK, and NFATc1. The groups stimulated for 1, 3, and 7 days displayed gradual increases in expression, and the highest expression level was detected on the seventh day (Fig. 3*F*). Moreover, the level of miR-134-5p in induced BMMs was determined by qRT-PCR. In contrast to osteoclast-related gene expression, the expression level of miR-134-5p showed gradual decreases after 1, 3, and 7 days of induction (Fig. 3*G*). These results confirmed that the combination of M-CSF and RANKL induces osteoclast differentiation.

### miR-134-5p antagomir accelerated osteoclast differentiation

**Figure 4:**
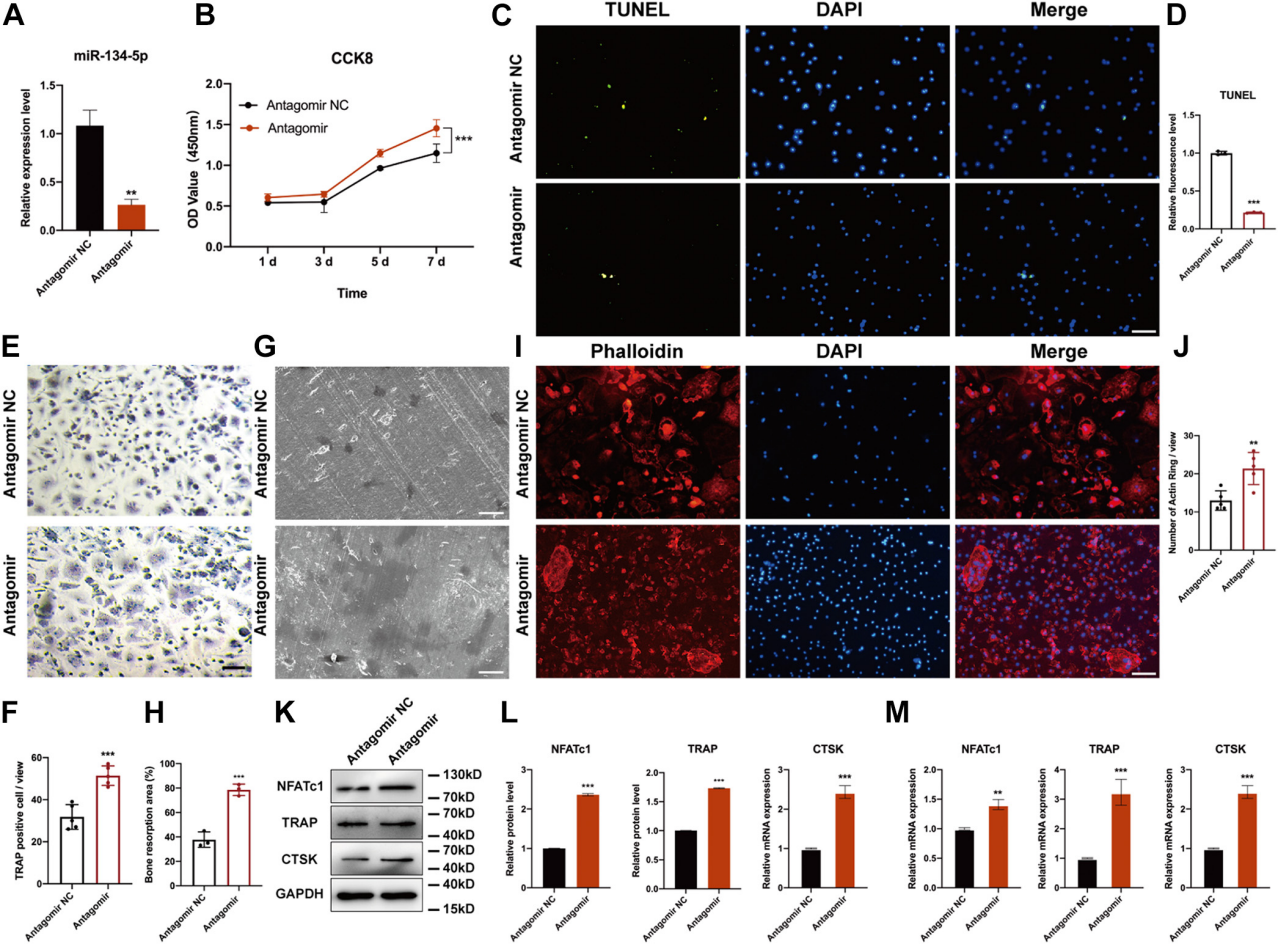
**miR-134-5p knockdown facilitates the osteoclast differentiation of BMMs.***A*, miR-134-5p antagomir or its negative control antagomir NC was transfected into BMMs, and the BMMs were then cultured with 30 ng/ml M-CSF and 50 mg/ml RANKL for 7 days to induce their differentiation into osteoclasts. The transfection efficiency was analyzed by qRT-PCR. *B*, the cell viability of BMMs transfected with antagomir or its control was determined by CCK-8 assay and measured under a microplate reader. *C* and *D*, cell apoptosis was determined with a TUNEL kit, and images were observed under a fluorescence microscope. The scale bars represent 100 μm. *E* and *F*, TRAP staining was performed for the analysis of osteoclast formation activity. The number of TRAP+ multinuclear osteoclasts was counted. The scale bars represent 100 μm. *G* and *H*, bone resorption ability was screened on bone slices through scanning electron microscope and bone resorption area was quantified with ImageJ software. The scale bars represent 50 μm. *I* and *J*, BMMs were induced to differentiate into osteoclasts and stained with phalloidin for the evaluation of actin ring formation. The number of F-actin rings per field was counted. The scale bars represent 100 μm. *K* and *L*, the protein expression of TRAP, CTSK, and NFATc1 was analyzed by Western blot. Semiquantitative analysis was conducted using ImageJ software. GAPDH was used as an internal reference control. *M*, quantitative RT-PCR was performed to measure the mRNA expression levels of TRAP, CTSK, and NFATc1. β-Actin was used as an internal reference gene. All data were expressed as means ± SD. ∗∗*p* < 0.01, ∗∗∗*p* < 0.001. BMMs, bone marrow macrophages; CCK-8, cell counting kit-8.

To investigate the role of miR-134-5p during osteoclastogenesis, we transfected miR-134-5p antagomir and miR-134-5p antagomir negative control (NC) into BMMs and then determined the knockdown efficiency by qRT-PCR. The antagomir and its negative control were successfully transfected (Fig. 4*A*). The cell viability after transfection with antagomir and its negative control was then measured by cell counting kit-8 (CCK-8) assays at 1, 3, 5, and 7 days. The absorbance value indicated that the knockdown of miR-134-5p accelerated cell proliferation (Fig. 4*B*). Moreover, cell apoptosis was detected with a TUNEL staining kit, and immunofluorescence images showed that miR-134-5p knockdown inhibited cell apoptosis compared with the negative control (Fig. 4, *C* and *D*). Subsequently, osteoclast differentiation was induced by combined treatment with M-CSF and RANKL for 7 days. TRAP staining was performed to identify the formation of mature osteoclasts. The number of TRAP-positive multinucleated cells in miR-134-5p antagomir was markedly higher than that found for the miR-134-5p antagomir NC group (Fig. 4, *E* and *F*). The regulation of miR-134-5p in bone resorption was measured in bone slices and bone resorption pits were detected by scanning electron microscopy. A higher number of bone lacunae was observed in the antagomir group than in the control group (Fig. 4, *G* and *H*). In addition, actin ring formation in osteoclasts was detected by phalloidin staining to better illustrate the function of miR-134-5p. Fluorescence microscope images revealed the same effects on actin ring formation in multinucleated cells as those observed with TRAP staining, which also showed an increasing number of osteoclasts (Fig. 4, *I* and *J*). For further confirmation, the protein and mRNA expression levels of TRAP, CTSK, and NFATc1 were determined. The expression levels of these genes were significantly higher in the miR-134-5p antagomir group (Fig. 4, *K*–*M*). Taken together, these data indicated that the miR-134-5p antagomir functions as a stimulus of osteoclast differentiation.

### miR-134-5p agomir inhibited osteoclast differentiation

**Figure 5:**
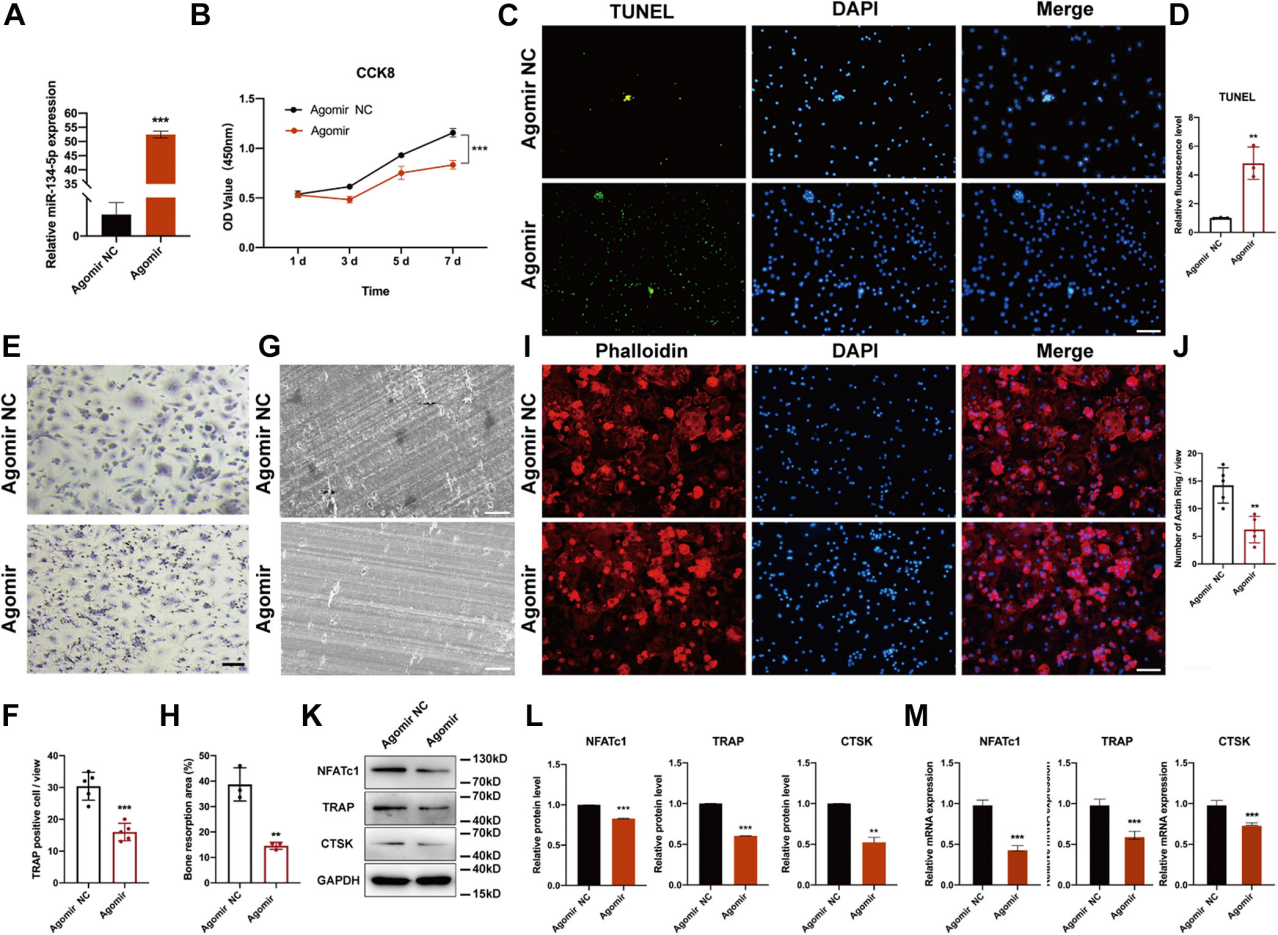
**miR-134-5p overexpression suppress the osteoclastogenesis process in BMMs.***A*, miR-134-5p agomir or its negative control agomir NC was transfected into BMMs, and the BMMs were then cultured in the presence of 30 ng/ml M-CSF and 50 mg/ml RANKL for 7 days. The transfection efficiency was analyzed by qRT-PCR. *B*, the cell viability of BMMs transfected with agomir or its negative control was detected by CCK-8 assay and measured at 450 nm under a microplate reader. *C* and *D*, cell apoptosis was determined with a TUNEL kit, and images were observed under a fluorescence microscope. The scale bars represent 100 μm. *E* and *F*, TRAP staining was used for the analysis of osteoclast activity. The number of TRAP-positive multinuclear osteoclasts was counted. The scale bars represent 100 μm. *G* and *H*, bone resorption ability was screened on bone slices through scanning electron microscope and bone resorption area was quantified with ImageJ software. The scale bars represent 50 μm. *I* and *J*, BMMs were induced to differentiate into osteoclasts and stained with phalloidin for the evaluation of actin ring formation. The number of F-actin rings per field was counted. The scale bars represent 100 μm. *K* and *L*, the protein expression of TRAP, CTSK, and NFATc1 was analyzed by Western blot. Semiquantitative analysis was conducted using ImageJ software. GAPDH was used as an internal reference control. *M*, the mRNA expression levels of TRAP, CTSK, and NFATc1 were analyzed by quantitative RT-PCR. β-Actin was used as an internal reference gene. All the data were expressed as the means ± SDs. ∗∗*p* < 0.01, ∗∗∗*p* < 0.001. BMMs, bone marrow macrophages; CCK-8, cell counting kit-8.

To further study the exact effect of miR-134-5p, we also transfected miR-134-5p agomir and miR-134-5p agomir NC into BMMs to determine whether miR-134-5p agomir could repress the process of osteoclast differentiation. The transfection efficiency was also determined by qRT-PCR, which showed an apparent increase in miR-134-5p after agomir transfection compared with that obtained after agomir NC transfection (Fig. 5*A*). Cell viability was then determined by CCK-8 after 1, 3, 5, and 7 days, and the results showed that the cells transfected with agomir exhibited a trend of decreasing viability compared with those transfected with the control agomir NC (Fig. 5*B*). Moreover, cell apoptosis was assessed using a TUNEL staining kit. The results were confirmed by images displaying more fluorescence staining in the agomir group compared with the corresponding group, and the relative fluorescence intensity of the agomir group was also higher than that obtained with the corresponding control (Fig. 5, *C* and *D*). After 7 days of induction, TRAP staining, bone resorption assay, and phalloidin staining were performed to explore the morphology and resorption function of mature osteoclasts. The number of TRAP-positive cells and bone resorption lacunae formation were significantly reduced in cells transfected with miR-134-5p agomir compared with those transfected with miR-134-5p agomir NC (Fig. 5, *E*–*H*). The actin ring formation was markedly decreased in the agomir group compared with the control group (Fig. 5, *I* and *J*). The expression profiles of TRAP, CTSK, and NFATc1 determined by Western blot and qRT-PCR suggested that the overexpression of miR-134-5p resulted in a marked reduction in the expression of osteoclast-related genes (Fig. 5, *K*–*M*). These data revealed that the miR-134-5p agomir inhibits osteoclastogenesis. Therefore, miR-134-5p negatively regulated osteoclast formation, as shown by an increase in miR-134-5p knockdown and a decrease in miR-134-5p overexpression.

### Itgb1 is a direct target of miR-134-5p

**Figure 6:**
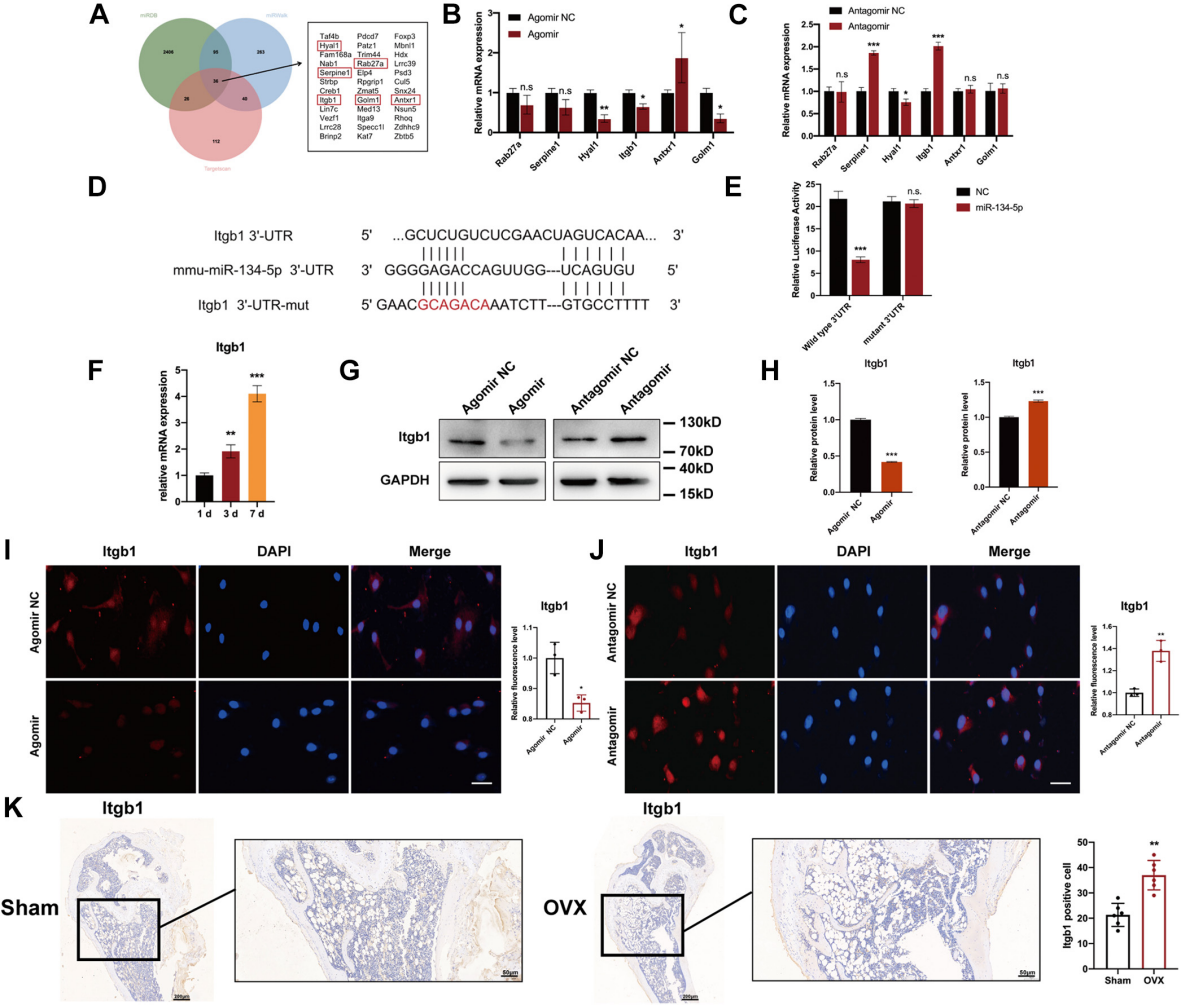
**Itgb1 is a direct target of miR-134-5p.***A*, diagram showing the potential targets of miR-134-5p predicted by TargetScan, miRDB, and miRWalk. Crosschecking of these data indicated six potential targets of miR-134-5p. *B* and *C*, the mRNA expression levels of Rab27a, Serpine1, Hyal1, Itgb1, Antxr1, and Golm1 in BMMs with miR-134-5p overexpression or knockdown were measured by qRT-PCR. β-Actin was used as an internal reference gene. *D*, bioinformatics prediction of miR-134-5p binding sites in the 3′-UTR of Itgb1 mRNA. *E*, the luciferase activities of a reporter containing the 3′-UTR of Itgb1 in miR-134-5p-transduced, miR-134-5p-mutant-transduced, or negative control 293T cells were determined. *F*, the mRNA expression level of Itgb1 was determined at 1, 3, and 7 days as BMMs were induced into osteoclasts. *β*-Actin was used as an internal reference gene. *G* and *H*, the protein expression levels of Itgb1 were determined by Western blot. *I* and *J*, immunofluorescence staining was performed to detect Itgb1 expression after miR-134-5p overexpression or knockdown. The relative fluorescence intensity was measured. The scale bars represent 25 μm. *Κ*, immunohistochemistry staining of sham and OVX mouse bone tissue was performed to evaluate Itgb1 expression *in vivo*. The number of Itgb1-positive cells was measured (n = 6). The scale bars for main panels represent 200 μm. The scale bars for the zoom magnification panels represent 50 μm. All the data are expressed as the means ± SDs. ∗*p* < 0.05, ∗∗*p* < 0.01, ∗∗∗*p* < 0.001. BMMs, bone marrow macrophages; OVX, ovariectomized.

miRNAs are associated with a large number of mRNAs, and miRNA–mRNA axes participate in various pathways regulating cell differentiation (22). To explore the potential genes regulated by miR-134-5p, we first searched TargetScan (http://www.targetscan.org), miRDB (http://www.mirdb.org/), and miRWalk (http://mirwalk.umm.uni-heidelberg.de) to identify potential targets of miR-134-5p. After crosschecking, a total of 36 potential genes were identified (Fig. 6*A*). Six genes that reportedly induce osteoclast differentiation were selected: Golm1, Rab27a, HYAL1, Serpine1, Antxr1, and Itgb1. The expression levels of these genes after the overexpression or knockdown of miR-134-5p in BMMs were detected. Itgb1 was downregulated after miR-134-5p overexpression but upregulated after miR-134-5p knockdown. The other five genes (Golm1, Rab27a, Hyal1, Serpine1, and Antxr1) did not display the same trend (Fig. 6, *B* and *C*). As a result of these analyses, Itgb1 was selected as a potential target of miR-134-5p, and a direct miR-134-5p-binding site in the 3′-UTR of Itgb1 mRNA was detected using prediction websites (Fig. 6*D*). Moreover, we conducted a luciferase reporter assay to confirm the interaction between miR-134-5p and Itgb1. We constructed two different types of luciferase reporter vectors, WT (Itgb1-wt) and mutant (Itgb1-mut), cotransfected them with miR-134-5p mimics and detected the luciferase activity by absorbance. As predicted, the luciferase activity of the WT was significantly reduced by miR-134-5p overexpression, whereas the mutant type showed no significant difference (Fig. 6*E*). The expression of Itgb1 was gradually increased at 1, 3, and 7 days to substantiate its regulatory function in osteoclastogenesis (Fig. 6*F*). To clarify the exact connection between miR-134-5p and Itgb1, we conducted Western blot to show that the protein level of Itgb1 was decreased by the miR-134-5p agomir and increased by the miR-134-5p antagomir compared with the negative control (Fig. 6, *G* and *H*). Immunofluorescence staining was conducted to clarify the expression level of Itgb1 in BMMs transfected with agomir or antagomir. The Itgb1 level was markedly higher in BMMs transfected with antagomir than in BMMs transfected with agomir (Fig. 6, *I* and *J*). In addition, the results from immunohistochemical staining *in vivo* were indicated. Higher Itgb1 expression levels and larger areas showing Itgb1 expression were detected in bone slices from femurs of OVX mice than those of sham mice (Fig. 6*K*). These findings demonstrated that Itgb1 is a direct target of miR-134-5p.

### miR-134-5p suppresses osteoclast differentiation by targeting Itgb1 and activating the MAPK pathway

**Figure 7:**
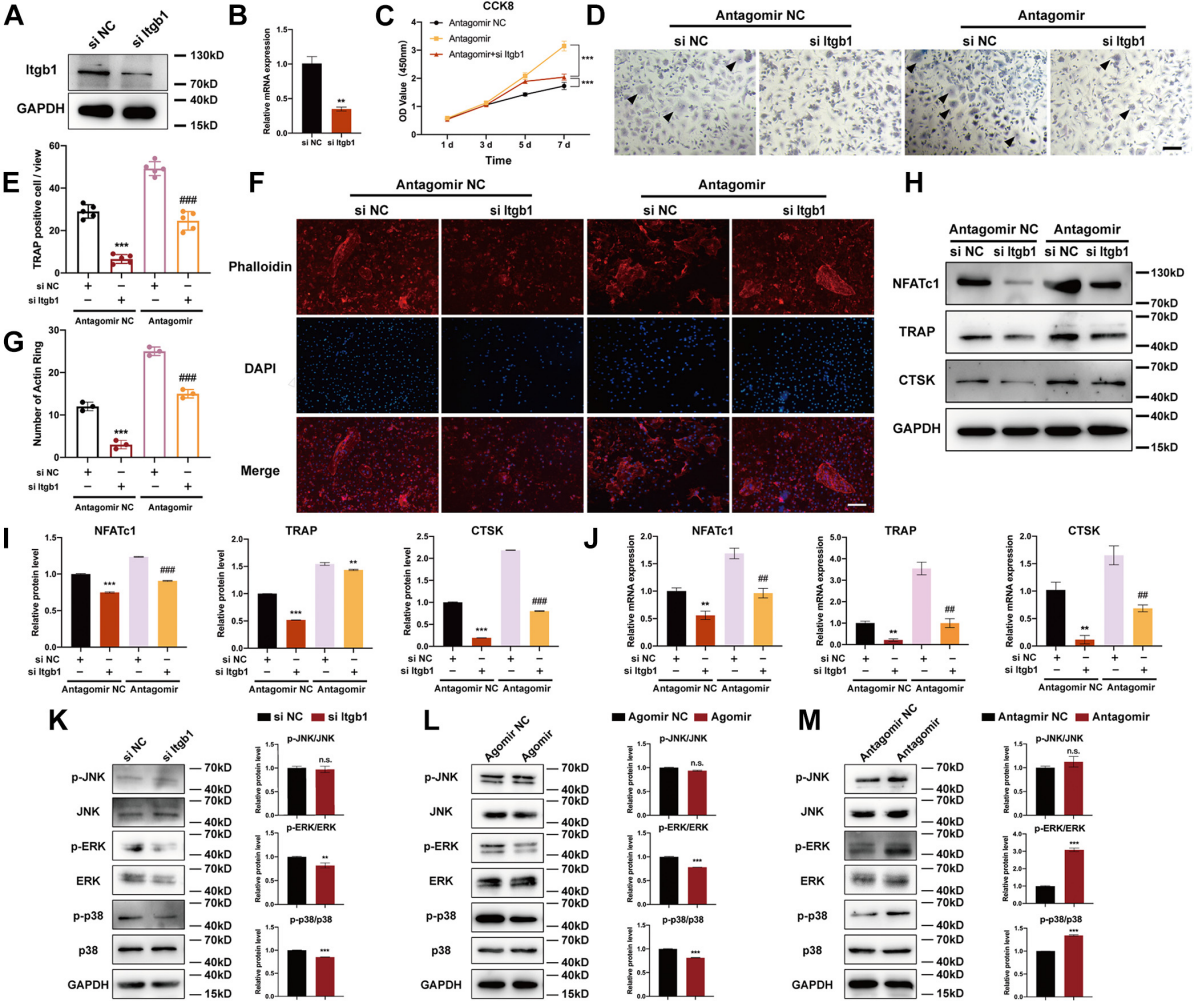
**miR-134-5p suppresses osteoclast differentiation by targeting Itgb1.***A* and *B*, BMMs were transfected with si Itgb1 or si NC, and the transfection efficacy was measured by Western blot and qRT-PCR. *C*, the cell viability of BMMs transfected with antagomir or its control along with si Itgb1 was determined by CCK-8 assay and measured under a microplate reader. *D* and *E*, BMMs were transfected with si Itgb1 or its negative control, subjected to antagomir or antagomir NC transfection, and then induced to differentiate into osteoclasts. The number of TRAP-positive multinuclear osteoclasts was counted *via* TRAP staining. The scale bars represent 100 μm. *F* and *G*, transfected BMMs were induced to differentiate into osteoclasts and stained with phalloidin. The number of F-actin rings per field was counted. The scale bars represent100 μm. *H* and *I*, the protein expression levels of TRAP, CTSK, and NFATc1 were determined by Western blot. The semiquantitative analysis of the protein expression levels of TRAP, CTSK, and NFATc1 is shown. GAPDH was used as an internal reference control. *J*, the mRNA expression levels of TRAP, CTSK, and NFATc1 in transfected BMMs were analyzed by quantitative RT-PCR. β-Actin was used as an internal reference gene. *K*–*M*, the protein expression levels of the phosphorylated of p38, ERK, and JNK among si Itgb1, agomir, and antagomir groups were determined by Western blot. Semiquantitative analysis of the p-p38/p38, p-ERK/ERK, and p-JNK/JNK ratios was shown. GAPDH was used as an internal reference control. All the data are expressed as the means ± SD. ∗∗*p* < 0.01 and ∗∗∗*p* < 0.001 compared with the antagomir NC+si NC group; ^##^*p* < 0.01 and ^###^*p* < 0.001 compared with the antagomir+si NC group. BMMs, bone marrow macrophages; CCK-8, cell counting kit-8.

To decipher the mechanism underlying the miR-134-5p interaction with Itgb1 in osteoclast formation, osteoclast differentiation with Itgb1 knockdown was performed. The transfection efficiency of Itgb1 siRNA (si Itgb1) was detected by Western blot and qRT-PCR (Fig. 7, *A* and *B*). The viability of BMMs cotransfected with antagomir and si Itgb1 was measured to determine the function of Itgb1 in miR-134-5p knockdown BMMs. si Itgb1 reversed the excessive cell proliferation induced by the antagomir (Fig. 7*C*). TRAP staining and phalloidin staining were performed with the groups transfected with antagomir or antagomir NC and cotransfected with si NC or si Itgb1, respectively. The TRAP staining results showed a significant increase in the number of TRAP-positive multinucleated cells in the miR-134-5p antagomir group; however, Itgb1 knockdown reversed the facilitation of osteoclast differentiation observed with the miR-134-5p antagomir. Moreover, si Itgb1 reduced the number of TRAP-positive osteoclasts in comparison with that found with the negative control (Fig. 7, *D* and *E*). Phalloidin staining revealed the similar effects. In brief, the number of actin rings that formed was reduced by the cotransfection of antagomir and si Itgb1 into BMMs; nevertheless, transfection with antagomir alone markedly upregulated the number of actin rings (Fig. 7, *F* and *G*). Western blot and qRT-PCR data showed that the miR-134-5p antagomir greatly increased the TRAP, CTSK, and NFATc1 expression levels, whereas si Itgb1 reversed the promoting effect of miR-134-5p antagomir on osteoclastogenesis (Fig. 7, *H*–*J*). All of these results suggested that miR-134-5p suppressed osteoclast differentiation by targeting Itgb1.

**Figure 8:**
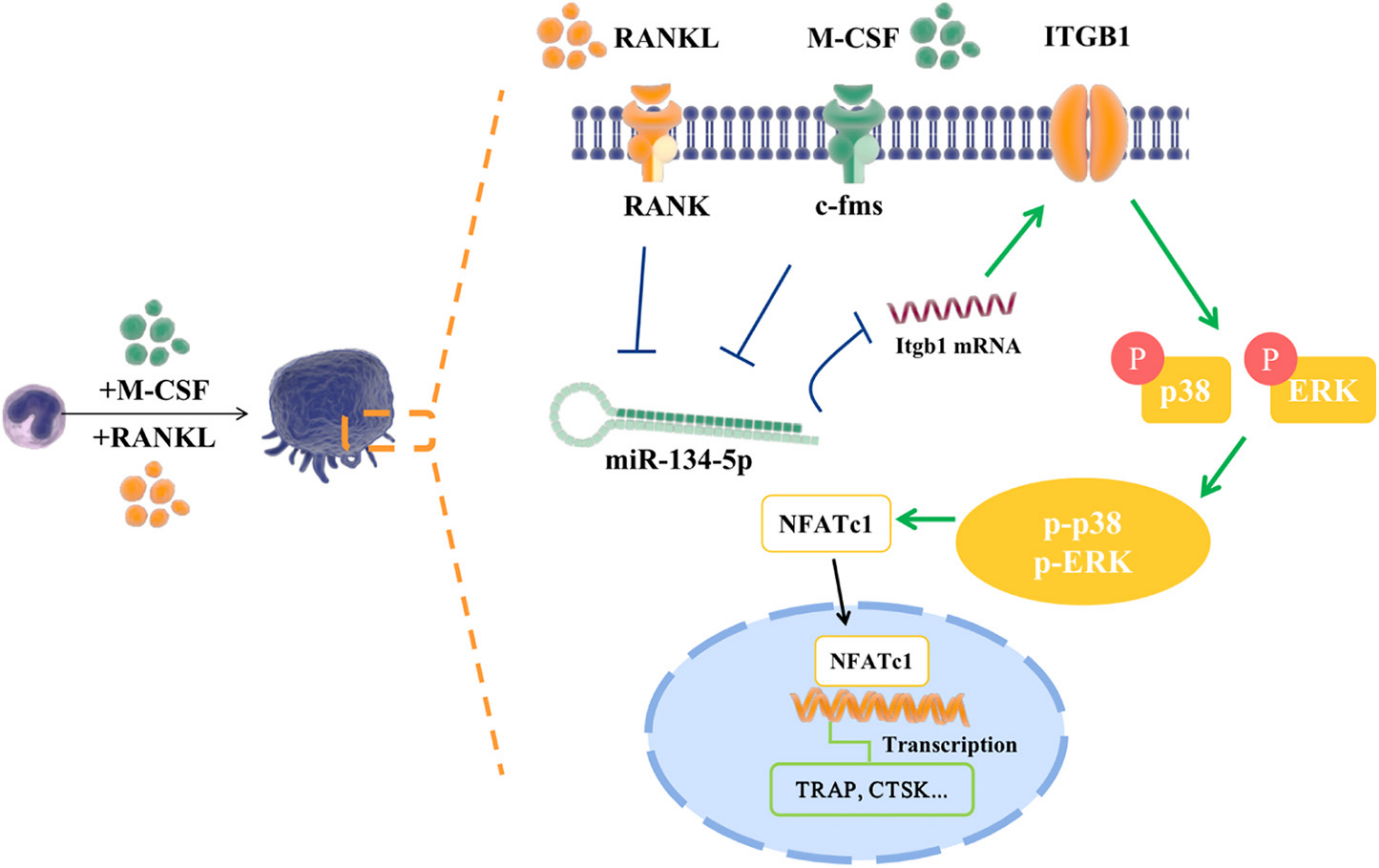
Schematic diagram of the involvement of the miR-134-5p/Itgb1/MAPK axis in osteoclast formation.

Having determined that miR-134-5p promotes osteoclast formation, we subsequently aimed to study the potential downstream pathway involved in this process. As indicated by the KEGG enrichment analysis, the miRNA–mRNA network was primarily related to the MAPK pathway (Fig. 2*E*). Moreover, the MAPK pathway has been proven to be crucial for bone metabolism and osteoclast differentiation (23). p38 and Erk and their phosphorylated forms p-p38 and p-Erk in BMMs transfected with agomir or antagomir alone or transduced with si Itgb1 or its negative control were detected. The protein level of p-p38 and p-ERK in BMMs transfected with Itgb1 siRNA or agomir were significantly decreased and that were increased in antagomir BMMs, as shown by Western blot, but the p-JNK expression showed no significant difference in siRNA, agomir, and antagomir groups (Fig. 7, *K*–*M*). Collectively, our data indicated that the regulation of osteoclast differentiation by miR-134-5p mainly occurs *via* the Itgb1/MAPK pathway (Fig. 8).

## Discussion

Our results revealed a novel role of the miR-134-5p/Itgb1 axis in regulating osteoclastogenesis. Using gene chip technology, we searched for miRNAs in OVX mice and discovered that miR-134-5p was significantly downregulated. The expression level of miR-134-5p was decreased in both bone and BMMs of OVX mice compared with sham mice, indicating a potential role in osteoclastogenesis. The same results were confirmed by *in vitro* experiments, which resulted that miR-134-5p significantly suppressed osteoclast differentiation by targeting Itgb1 and highlight that Itgb1 is an underlying target in the bone remodeling process.

Osteoclastogenesis initiates with the recruitment of osteoclast precursors, during control of the activation of several osteoblast-related factors activation, such as RANKL, OPG, and Semaphorin 3A (24). In this phase, factors such as RANKL act *via* the RANK receptor, leading to the production of TRAF6. These mutual effects activate NF-κB and other pathways and then induce the transcription of nuclear factor of activated T cells c1 (NfATc1), which results in the initiation of osteoclast differentiation (25, 26). miRNAs are a type of noncoding endogenous RNA that function as posttranscriptional regulators of genes (27). Several types of RNases process pre-miRNA molecules into mature miRNAs, which bind to the 3′-UTR of target mRNAs to recognize and silence target genes (28). Among the multiple types of regulatory factors, miRNAs have been identified as a valid tool for regulating osteoclast formation and bone resorption. miR-335 and miR-338 reportedly negatively regulate RNAKL in osteoclast precursors, whereas in osteoblasts, miR-145 induce OPG expression to decrease the RANKL/OPG ratio, leading to the inhibition osteoclastogenesis (29, 30, 31). In this study, we first screened for downregulated miRNAs through the microarray analysis, and miR-134-5p was one of the significantly downregulated miRNAs. As a member of the miRNA family, miR-134-5p has been found to participate in cell biological functions and metabolism, such as cell invasion (32) and metastasis (33, 34). In addition, this miRNA is involved in mediating the behavior and progression of cancer cells, such as breast cancer cells (35). However, the role of miR-134-5p in bone homeostasis still requires clarification. Although miR-134-5p enables phosphate-induced calcium deposition in vascular smooth muscle cells (18), there is no evidence showing its exact function in bone formation and resorption. Therefore, in this study, we detected miR-134-5p expression was decreased in the osteoclast formation process *in vitro* and in OVX mice *in vivo*. Based on these results, the effects of this miRNA were verified by the overexpression or knockdown of miR-134-5p. The results showed that the knockdown of miR-134-5p accelerated osteoclast formation and that its overexpression inhibited this process. The findings confirmed that miR-134-5p is a negative mediator of osteoclastogenesis.

miRNAs mediate osteoclast differentiation and formation by negatively controlling downstream genes (21). miR-148a was confirmed to induce osteoclast formation through inhibition of MAFB, a repressor of the transcription factor NFATc1 (36). Chen *et al.* (37) described that miR-503 negatively regulates RANK, which is the receptor of RANKL in activating osteoclastogenesis. miR-128 has been shown to affect osteoclast formation by targeting SIRT1, which is associated with postmenopausal osteoporosis (38). Additionally, miR-21-5p regulates macrophage migration and osteoclast differentiation *via* SKP2 function (39). In our study, the prediction of potential target genes of miR-134-5p was performed using online prediction websites, and crosschecking showed that several transcripts involved in osteoclastic progression, namely, Golm1, Rab27a, Hyal1, Serpine1, Antxr1, and Itgb1. Denais *et al.* discovered a novel secretion pathway protein, Golm1, that interacts with dymeclin to influence endochondral bone formation and Shen *et al.* found that Golm1 could promote osteoporosis through inhibition of the osteogenic differentiation of BMSCs (40, 41). Rab27a interference rescued dysfunctional osteoblasts originating from OVX mice and was restrained by miR-124 to attenuate osteoclastogenesis (42, 43). Moreover, previous studies have shown that Hyal1 expression is upregulated *via* osteoclast formation and that Serpine1 is enriched in osteoporosis patients (44, 45). In addition, Antxr1 is also involved in RANKL-induced osteoclastogenesis (46).

The integrin family, which includes heterodimeric transmembrane molecules that can bind the extracellular matrix, is known as a crucial regulator of cell adhesion, cell survival, and differentiation in different cell lines (47, 48, 49). Numerous studies have revealed the regulatory effect of integrin β1 on chondrogenesis (50), neurological behavior and neurovascular regeneration (51), and lung tissue homeostasis (52). Studies have investigated the function of Itgb1 in facilitating osteoclast differentiation (53). Wang *et al.* (54) discovered that during osteoclastogenesis, OVX Itgb1-depleted mice exhibit less bone loss than sham mice. α9/β1 integrin accelerates increases in osteoclast activity during inflammatory bone resorption (55)^.^ In addition, in adjuvant-induced arthritis rats, miR-124 attenuates osteoclast formation by targeting NFATc1 and Itgb1, and immunohistochemistry analysis has revealed that Itgb1-positive cells share the same position as TRAP-positive cells (56). We hypothesized that miR-134-5p potentially binds to the 3′-UTR of Itgb1. We then performed luciferase reporter assays to verify the relationship between miR-134-5p and Itgb1. The mRNA and protein expression profiles of Itgb1 indicated that Itgb1 was directly regulated by miR-134-5p. To determine the function of Itgb1 in osteoclastogenesis, we designed a rescue experiment with miR-134-5p antagomir transfection. The results showed that the deletion of Itgb1 resulted in a decrease in the osteoclast number and the cotransfection of BMMs with miR-134-5p antagomir, and si Itgb1 inhibited osteoclast formation compared with that obtained with transfection with miR-134-5p antagomir alone.

Accumulating evidence has established the role of MAPK signaling in many fields of cellular activity and metabolism, including osteoclastogenesis. MAPK signaling is mainly activated by TRAFs, an adapter protein that is recruited by RANK binding with RANKL (57). The initiation of MAPK is essential for the activation of downstream factors, including NFATc1 and c-Fos (58). Studies have also shown that p38α-deficient mice are more likely to develop osteoporosis and exhibit a marked decrease in bone mass (59). In addition, osteoclast precursors are relevant to the switch from cell proliferation to differentiation by activating the MAPK pathway (60). Moreover, osteoclasts can perceive various extracellular matrices through integrins, which enhances osteoclast formation. Recent studies have reported that Itgb1 shares a regulatory function with MAPK pathway signaling in osteoclastogenesis. Osteoclastogenesis is suppressed by the integrin β1/FAKpY397/MAPK pathway on a titanium implant surface (23). In our present study, a KEGG enrichment analysis of OVX mice revealed that miRNAs were mainly related to the MAPK pathway, underlying a potential miR-134-5p/Itgb1 network involved in osteoclast differentiation through the MAPK pathway. Hence, we focused on the integrin β1 and MAPK relationship to investigate the profound mechanism through which miR-134-5p regulates osteoclast formation. The protein expression levels of phosphorylated-p38 (p-38), phosphorylated-ERK (p-ERK), and phosphorylated-JNK (p-JNK) in miR-134-5p agomir or antagomir-transfected BMMs and in BMMs transduced with si Itgb1, were determined by Western blot, and the results indicated that the knockdown of miR-134-5p increased p-p38 and p-ERK expression and that these molecules were decreased after miR-134-5p overexpression. The p-p38/p38 and p-ERK/ERK ratios were used to determine the degree of activation in osteoclastogenesis, and the increasing tendencies in BMMs transfected with antagomir demonstrated a facilitation of osteoclast differentiation. Thus, the osteoclast differentiation process was initiated. Because Itgb1 is known as an upstream regulator of the MAPK pathway, the corresponding changes in Itgb1 silenced BMMs showed decreases in the expression level of MAPK pathway, which illustrated the regulatory impact of Itgb1 on MAPK. MAPK members exhibited the same decline as Itgb1 after miR-134-5p was overexpressed and were increased with miR-134-5p knockdown, further explaining the involvement of miR-134-5p in regulating osteoclastogenesis through MAPK pathway. These results demonstrated that the MAPK pathway was involved in miR-134-5p regulated osteoclastogenesis through direct activation of the miR-134-5p target Itgb1.

miRNAs are extensively used to treat some pathological conditions, such as cancer, psoriasis, and cardiovascular disease, but no such applications have been found with respect to bone destruction activities (61). Because many contributors have been implicated in bone homeostasis, a single genetic error would convert the current environment into a pathological environment. Moreover, compensatory factors and mechanisms could be found to target these genetic errors and translate these elements into novel clinical approaches for diseases. Given the regulatory role of mRNAs of miRNAs in osteoclastogenesis, targeting miRNAs could be a promising therapeutic strategy for bone disorders, including osteoporosis. The downregulation of miR-134-5p in mice displaying osteoporotic features and in cells induced into osteoclasts sheds new light on the treatment of osteoporotic diseases. This miRNA may serve as a positive target for the inhibition of osteoporosis development.

In conclusion, the present study indicated that miR-134-5p suppressed osteoclast differentiation and inhibited bone resorption by downregulating the Itgb1/MAPK axis (Fig. 8). miR-134-5p was downregulated both in mice with osteoporosis and in osteoclasts cultured *in vitro*. The knockdown of this miRNA upregulated the expression of its negative target Itgb1 and induced an auxoaction in osteoclast differentiation. Therefore miR-134-5p/Itgb1 may be a potential target in the regulation of bone remodeling and osteoporosis therapy.

## Experimental procedures

### Animals

C57BL/6N mice (aged 4–8 weeks) were purchased from Beijing Vital River Laboratory Animal Technology. All the mice were housed in a standard animal facility under controlled temperature (21 °C) and photoperiod (12 h light/12 h dark) conditions with adequate water and food. All experimental procedures were approved by the medical ethics committee of the Laboratory Animal Center of Beijing Institute of Basic Medical Sciences.

### Construction of an OVX mouse model

Eight-week-old female mice (n = 12) were randomly divided into the sham-operated (sham) (n = 6) or OVX (n = 6) groups. The bilateral ovaries of the OVX group were removed from an incision on the back, whereas those of the sham group were subjected to laparotomy without ovariectomy as previously described (62). In brief, the mice were anesthetized by i.p. injection of pentobarbital sodium (10 mg/kg body weight) and placed in the prone position. The hair on the surgical area was cut on the back, the skin was sterilized and cut, and the muscle was separated bluntly. The bilateral ovaries were stripped and removed after the uterus was ligated to stop the bleeding. The muscle layer and skin incision were sutured, and penicillin was injected to prevent infection. All the animals were bred under the same living environment with the same food and environmental conditions. After ovariectomization, the body weights of all the mice were determined every week for 8 weeks. Eight weeks later, the mice were sacrificed, and their femurs were harvested for further experiments.

### Micro-CT scanning

After 8 weeks, femur bones were harvested, fixed for 48 h in 4% paraformaldehyde, and analyzed using a micro-CT imaging system (Quantum GX, PERKinElmer). The scanning parameters were set as follows: equidistant definition, 10 mm; X-ray energy settings, 70 kV and 70 mA; and a voxel size of 10 mm in 3D form. After reconstruction, a region of interest was selected for further measurements. For the trabecular instruction investigation, we detected the bone volume to tissue volume (%), trabecular number (1/mm), trabecular seperation/spacing (millimeter), and trabecular thickness (millimeter).

### Histological analysis by H&E and immunohistochemical staining

After 8 weeks, the mice in each group were sacrificed *via* i.p. overdosing injection, and the femurs were removed for histological analysis. The femur samples were fixed in 4% paraformaldehyde for 24 h, decalcified in 10% EDTA for 4 weeks, dehydrated, diaphanized, and embedded in paraffin. The processed samples were sectioned into 4 μm slices and stained with H&E (Sigma–Aldrich) following the manufacturer’s instructions.

For immunohistochemistry analysis, TRAP and Itgb1 were used to quantify the number and area of osteoclasts. Briefly, the samples were cooked in a pressure cooker for 3 min and 30 s with antigen retrieval solution, cooled at room temperature (RT), and incubated with 3% hydrogen peroxide for 10 min. The samples were then incubated overnight at 4 °C with primary antibody and then with secondary antibody. According to the manufacturer’s instructions, 3,3-diaminobenzidine tetrahydrochloride chromogenic agent was prepared, and target slides were then incubated with the agent for 5 min. Subsequently, the samples were counterstained with hematoxylin (Abcam), dehydrated, and mounted.

### Microarray analysis

Total RNA was extracted from the tibias and femurs of sham and OVX mice with TRIzol reagent (Sigma–Aldrich). Microarray analysis was then performed using TruSeq Small RNA Library Preparation Kits (Illumina) to provide reagents for the generation of small RNA libraries directly from total RNA, and RNA-Sequ was performed on an Illumina HiSeq platform (Illumina). The raw sequencing reads were calculated through a comparative analysis using Bowtie (version 1.2.3, http://bowtie-bio.sourceforge.net/bowtie2) and Circos (version 0.69-6, http://circos.ca/). To identify the differentially expressed miRNAs, |log2FC| ≥ 1 and *p* value < 0.05 were used as the default parameters. A KEGG pathway enrichment analysis was then performed using Goatools (version 0.6.5). The final visualization of heatmaps was performed using R v3.6.0 software (https://mirrors.tuna.tsinghua.edu.cn/CRAN/).

### Isolation of BMMs, cell culture, and differentiation

For osteoclastogenesis assays, we isolated BMMs from 6-week-old C57BL/6N mice and cultured them in complete minimum essential medium eagle alpha modifications (α-MEM) medium with M-CSF (30 ng/ml) and RANKL (50 ng/ml) for 7 days as described previously (63). In brief, the femurs and tibias of 6-week-old mice were surgically removed, and the bone marrow was flushed out with warm, serum-free α-MEM medium using a 1 ml syringe. The resultant substrate was centrifuged at 1000 rpm for 5 min, and the resuspension was cultured in α-MEM containing 10% fetal bovine serum (Gibco) with 30 ng/ml M-CSF (PeproTech) for 3 days to form osteoclast precursors.

For *in vitro* osteoclast differentiation, we cultured BMMs (1 × 10^6^ cells/well) in a 24-well plate in α-MEM supplemented with RANKL (50 ng/ml) and M-CSF (30 ng/ml). The cells were cultured for 7 days, and the medium was changed every 2 days.

### TRAP staining

The cells were fixed in 4% paraformaldehyde for 20 min, stained using a diagnostic acid phosphatase staining kit for TRAP analysis (Sigma), photographed to detect TRAP+ multinucleated cells (possessing more than three nuclei), analyzed to determine the percentage of TRAP+ multinucleated cells, and counted under an inverted microscope.

### Analysis of actin ring formation

Cells from each group were fixed in 4% paraformaldehyde for 15 min at 4 °C, permeabilized with 0.1% Triton X-100 for 10 min at RT, and incubated with rhodamine-phalloidin (Invitrogen) for 30 min. After three washes with PBS, the cells were incubated with DAPI (Sigma–Aldrich), washed, and observed under a fluorescence microscope (Leica).

### qRT-PCR

Total RNA was extracted from bone tissue or cells in each group using a transcriptase kit (Toyobo) with TRIzol reagent according to the manufacturer’s instructions, and total RNA was quantified using a NanoDrop 2000 (Thermo). Subsequently, 1 μg of total RNA was reverse-transcribed into complementary DNA (cDNA), and the resulting cDNA was used as the template in qRT-PCR with SYBR Green (Toyobo) for the assessment of specific gene expression. miR-134-5p detection was performed using miRNA First Strand cDNA Synthesis (Tailing Reaction) and MicroRNAs qPCR Kit (SYBR Green Method) (Sangon Biotech) according to the instructions. The reactions were run in triplicate. The relative mRNA or miRNA levels were normalized to those of endogenous β-actin or U6. The expression levels were calculated using the 2^−ΔΔCt^ method to determine the fold changes in expression between the experimental and control groups. The miRNA and mRNA primer sequences used in this study are listed in Table 1.

**Table 1:** Primer sequences

Gene	Primer	Sequences(5′-3′)
TRAP	Forward	CAAGAACTTGCGACCATTGTTA
	Reverse	ATCCATAGTGAAACCGCAAGTA
CTSK	Forward	GCTTGGCATCTTTCCAGTTTTA
	Reverse	CAACACTGCATGGTTCACATTA
NFATc1	Forward	GAGAATCGAGATCACCTCCTAC
	Reverse	TTGCAGCTAGGAAGTACGTCTT
Itgb1	Forward	TACTCTGGAAAATTCTGCGAGT
	Reverse	ATAGCATTCACAAACACGACAC
miR-134-5p	Forward	TATGTGACTGGTTGACCAGAGGGG
	Reverse	GTGCAGGGTCCGAGGT
Runx2	Forward	CCTTCAAGGTTGTAGCCCTC
	Reverse	GGAGTAGTTCTCATCATTCCCG
ALP	Forward	TCATTCCCACGTTTTCACATTC
	Reverse	GTTGTTGTGAGCGTAATCTACC
OPN	Forward	AAACACACAGACTTGAGCATTC
	Reverse	TTAGGGTCTAGGACTAGCTTGT
Antxr1	Forward	GCACCACTGGAATGAAATCTAC
	Reverse	TTCCCTGTCCTCAGTTAGTTTC
Serpine1	Forward	ATCTTGGATGCTGAACTCATCA
	Reverse	GAGAGAACTTAGGCAGGATGAG
Hyal1	Forward	TCACACATTCCAGGACATCAAG
	Reverse	CCACTAAAGTTTCTGGCCAATC
Golm1	Forward	TTCCAATTCCAGAAGAACCAGACCAG
	Reverse	TCCTCTATCCGCTCGTCACACTG
Rab27a	Forward	CGGAATCCCCTATTTTGAAACC
	Reverse	CTTCTCCTTCTCCTCACTTAGC
β-Actin	Forward	TCACTATTGGCAACGAGCGGTTC
	Reverse	CAGCACTGTGTTGGCATAGAGGTC
U6	Forward	GCGCGTCGTGAAGCGTTC
	Reverse	GTGCAGGGTCCGAGGT

### Cell transfection

To knock down or overexpress miR-134-5p, BMMs were transfected with miR-134-5p agomir, miR-134-5p antagomir, or the corresponding negative controls (5 nM; Sangon) for 24 h using Lipofectamine 2000 (Invitrogen) according to the manufacturer’s instructions. The transfection efficiency was analyzed by qRT-PCR.

For gene knockdown, mouse Itgb1 and control siRNAs were purchased from HanBio. BMMs were transfected with 40 nM siRNA. The medium was replaced 6 h later with α-MEM containing 10% fetal bovine serum and 30 ng/ml M-CSF. After 48 h, the efficiency of transfection was determined by Western blot and qRT-PCR. The transfected BMMs were then treated with M-CSF and RANKL for further experiments.

### Cell viability assay

Isolated BMMs were seeded in 96-well plates at a density of 2 × 10^5^ cells per well in α-MEM supplemented with M-CSF (30 ng/ml) for 3 days. After transfection with agomir, antagomir, and their negative controls, cells were incubated with 10 μl of CCK-8 (Beyotime) combined with fresh α-MEM medium for 1, 3, 5, and 7 days. The optical density at 450 nm was then measured using a microplate reader (Thermo Multiskan FC) to reflect the cell viability. The experiment was repeated three times.

### TUNEL assay

After transfection with agomir, antagomir, and their corresponding negative controls, BMMs were fixed with 4% paraformaldehyde for 30 min and washed with PBS. The BMMs were then incubated with 0.3% Triton X-100 in PBS at RT and washed twice with PBS. According to the manufacturer’s instructions, the detection reagent was prepared, and the cells were incubated with the agent at 37 °C in the dark. The fluorescence staining was measured under a fluorescence microscope.

### Bone resorption assay

BMMs were seeded on bone slices at a density of 1 × 10^6^ cells per well and induced with 30 ng/ml M-CSF and 50 ng/ml RANKL for 7 days. After induction, the cells were removed with 2% hypochlorite solution and washed three times with PBS. The slices were then scanned under an electron microscope, and the pit areas were quantified using ImageJ software (https://imagej.net/).

### Western blot analysis

The cells were first washed three times with PBS and lysed with radioimmunoprecipitation assay buffer with complete protease inhibitor (Solarbio). After protein quantification, the samples were separated by 12% SDS-PAGE and transferred to polyvinylidene difluoride membranes (Bio-Rad) according to the standard protocol. The membranes were blocked with 5% skim milk and incubated overnight at 4 °C with the appropriate primary antibodies. The primary antibodies were as follows: anti-TRAP, anti-CTSK, anti-NFATc1, and anti-Itgb1 (1:1000; Cell Signaling Technology). β-Actin was obtained from Affinity Biosciences. The membranes were subsequently incubated with secondary antibodies for 1 h at RT and washed with a Tris-buffered saline with Tween-20 for 30 min. The blots were visualized using Tanon image scanning. ImageJ software was used for quantitative analysis of the blots.

### Target prediction

To explore the explicit mechanism through which miR-134-5p affected osteoclast formation, we used three separate databases to predict potential miR-134-5p target genes: TargetScan, miRDB, and miRWalk.

### Luciferase reporter assays

The interactions between miR-134-5p and Itgb1 were confirmed by a luciferase reporter assay. The sequences that contained the WT (Itgb1-wt) or mutant (Itgb1-mut) seed region were synthesized and cloned into a luciferase reporter plasmid. For the detection of repression by miR-124, host 293T cells were cotransfected with the indicated reporter construct and a Renilla luciferase plasmid. Twenty-four hours after transfection, the luciferase activities were measured using a fluorescence spectrophotometer (Thermo Multiskan FC) according to the manufacturer’s instructions. The relative transcriptional activity was normalized to the corresponding vehicle control value.

### Immunofluorescence analysis

For immunofluorescence analysis, the cells seeded on slides were fixed in 4% paraformaldehyde for 20 min at RT and permeabilized with 0.1% Triton X-100 for 5 min. The slides were then incubated overnight at 4 °C with a primary antibody against Itgb1. Thereafter, the cells were stained with secondary antibody at RT in the dark for 2 h. The nuclei were stained with DAPI for 5 min and imaged under a laser scanning confocal microscope (Leica).

### Statistical analysis

Statistical analysis was performed using SPSS software (version 20.0.0.0, https://www.ibm.com/cn-zh/analytics/spss-statistics-software) and GraphPad Prism 7.0 (GraphPad Software). The quantitative data are expressed as the means ± SDs. A statistical analysis of the differences between groups was performed by two-tailed unpaired Student’s *t* test. One-way ANOVA was used for comparisons among more than three groups. A *p* < 0.05 was considered significant.

## Conclusion

In conclusion, the present study indicated that miR-134-5p suppressed osteoclast differentiation and inhibited bone resorption by downregulating the Itgb1/MAPK axis (Fig. 8). miR-134-5p/Itgb1 may be a potential target in regulating bone remodeling and osteoporosis therapy.

## Data availability

All the data indicated in this study are available upon request by contact from the corresponding author.

## Conflict of interest

The authors declare that they have no conflicts of interest related to the contents of this article.
